# *Ageratina adenophora* aqueous extract impairs *Ascaris suum* egg embryonation and infectivity via disruption of metabolic and detoxification pathways

**DOI:** 10.3389/fvets.2026.1789026

**Published:** 2026-04-22

**Authors:** Luyang Wang, Wenya Wang, Junjun He, Fanfan Shu, Jun Ma, Jianfa Yang, Fengcai Zou

**Affiliations:** 1 The Yunnan Key Laboratory of Veterinary Etiological Biology, College of Veterinary Medicine, Yunnan Agricultural University, Kunming, Yunnan, China; 2 College of Veterinary Medicine, Yunnan Agricultural University, Kunming, China

**Keywords:** *Ageratina adenophora*, *Ascaris suum* eggs, cytochrome P450, metabolism, transcriptomics

## Abstract

*Ageratina adenophora* is a globally invasive perennial herb that poses a significant threat to biodiversity. However, recent studies highlight its potential for ecological utilization, demonstrating antibacterial, anti-inflammatory, and insecticidal properties. Research on its effect on *Ascaris suum* eggs and the underlying molecular mechanisms remains limited. This study investigated the impact of *A. adenophora* aqueous extract on *A. suum* egg development and explored key responsive pathways and genes via RNA sequencing (RNA-Seq). *A. suum* eggs were treated *in vitro* with various concentrations (0.10%, 0.25%, 0.50%, 1.00%, 2.50%, 5.00%) of the extract. Embryonation rates decreased dose-dependently, with the 5.00% treatment showing the strongest inhibition (34.24%). Eggs treated with 2.50% and 5.00% extract caused significantly less severe pulmonary lesions and lower larval counts in infected mice, indicating reduced infectivity. Transcriptome analysis of control (WH), 0.10% (EH), and 0.50% (MH) treated eggs identified 281 and 1,083 differentially expressed genes (DEGs) for EH vs. WH and MH vs. WH, respectively. GO and KEGG enrichment analyses revealed that DEGs were primarily involved in transmembrane transport, catalytic activity, carbohydrate metabolic processes, the drug metabolism-cytochrome P450 pathway, and glycolysis/gluconeogenesis. Key genes, including CYP44A1, CYP4V2, CYP4C3, and GST-4, were significantly downregulated. qRT-PCR validated the RNA-Seq results. These findings suggest that *A. adenophora* extract inhibits *A. suum* egg development, potentially by disrupting energy metabolism and xenobiotic detoxification pathways. This study provides a theoretical basis for developing *A. adenophora*-based agents targeting the egg stage of *A. suum*.

## 1 Introduction

*Ageratina Adenophora*, commonly known as Crofton weed, is a perennial herb of the *Asteraceae* family. Originally introduced as an ornamental plant in the 1940s, it has since become a notorious invasive species in many tropical and subtropical regions worldwide, including southern China, particularly widely distributed in Yunnan Province (1). Its high adaptability and competitiveness enable rapid spread, which extensively disrupts ecosystem stability, threatens local biodiversity and agricultural productivity, and harms animal health, resulting in significant ecological and economic losses (2, 3). For instance, pollen and volatile organic compounds released by *A. adenophora* can induce allergic responses in humans, including respiratory irritation and dermal inflammation (4, 5). Furthermore, the plant contains toxic compounds such as tremetone, which when ingested by livestock may cause anorexia, weight loss, hepatic injury, and in severe cases, mortality (6, 7). These effects not only compromise livestock health but also reduce pasture availability and forage resources, ultimately undermining livestock production economics. Consequently, *A. adenophora* was listed as the top invasive species in China’s first official list in 2003 (8).

Given its escalating threat, researchers have increasingly explored strategies to transform this “harmful species” into a “utilizable resource,” aiming to mitigate ecological pressure and promote sustainable ecological, economic, and social development. Recent studies on *A. adenophora* have revealed that the plant contains a variety of secondary metabolites, including terpenoids, flavonoids, phenols, and alkaloids (9, 10). Notably, *A. adenophora* extracts have shown promising insecticidal and antiparasitic properties against a range of pests and parasites, such as ticks, mites, nematodes, and mollusks (11–14). This suggests its potential as a source of eco-friendly botanical pesticides, offering a strategy to transform an ecological threat into a valuable resource.

Ascariasis caused by the porcine roundworm *Ascaris suum* is a prevalent parasitic disease with global distribution, leading to significant economic losses in swine production due to reduced growth and feed efficiency (15, 16). During the larval stage, roundworms can migrate through the lungs, leading to larval ascariasis, which causes hemorrhage, edema, and inflammatory responses in the host’s lung tissue (17, 18). In industrialized countries, the epidemiological relevance of *A. suum* has increased in the context of animal welfare-oriented, cage free, and organic livestock farming systems for pigs (19). Furthermore, *A. suum* poses a zoonotic risk, highlighting its public health importance (16, 20). Control primarily relies on anthelmintics, but issues of drug resistance and residues necessitate the search for alternative agents. While studies have reported the anthelmintic effects of various plant extracts, including *A. adenophora*, against adult worms or other parasites, the specific impact of *A. adenophora* on *A. suum* eggs and the molecular mechanisms involved are poorly understood. Transcriptomics provides a powerful tool to elucidate these mechanisms at the gene expression level. Based on this, this study aimed to evaluate the effect of the aqueous extract of *A. adenophora* on the development and embryonation of *A. suum* eggs *in vitro*, assess the infectivity of the treated eggs in a mouse model, and identify key responsive genes and pathways through RNA-Seq analysis. The findings are expected to provide insights for developing *A. adenophora*-based anthelmintics and understanding its mode of action.

## 2 Materials and methods

### 2.1 Plant material and extract preparation

*A. adenophora* whole plants were collected in Yunnan Agricultural University, Kunming, Yunnan Province, China, in September 2023. The morphological identification of the plant was performed based on the taxonomic key. The extraction method of water extract from *A. adenophora* is as previously described (12). Leaves and stems were washed, air-dried, and ground into powder. A total of 500 g of powder was soaked in 2 L of distilled water at 60 °C overnight. The mixture was filtered sequentially through 60 and 200-mesh sieves. The filtrate was concentrated using a rotary evaporator at 60 °C to obtain a paste, which was then reconstituted with ultra-pure water to a stock solution of 1 g/mL (w/v), representing 1 gram of plant material per milliliter of water. The stock solution was stored at 4 °C in the dark. The plant specimen was identified morphologically based on taxonomic keys; however, a voucher specimen was not deposited in a herbarium. Chemical characterization of the aqueous extract was not performed in this study, which is a limitation.

### 2.2 *Ascaris suum* eggs

Adult *A. suum* worms were collected from the intestines of pigs at a slaughterhouse in Mengzi, Yunnan Province. They were transported to the laboratory in physiological saline and stored at 4 °C. Eggs were harvested from gravid female worms by dissecting the uteri, homogenizing in phosphate-buffered saline (PBS), filtering, and treating with 0.5% sodium hypochlorite for surface sterilization, following established protocols (21). The *A. suum* egg concentration was adjusted to 2.5 × 10^4^ eggs/mL in 0.1 N H_2_SO_4_ for cultivation.

### 2.3 *In vitro* treatment of *A. suum* eggs with *A. adenophora* extract

1.5 × 10^5^ *A. suum* eggs suspensions were added to 18 cm Petri dishes. The *A. adenophora* stock solution was diluted in 76 mL of 0.1 N H_2_SO_4_ to final concentrations of 0.10, 0.25, 0.50, 1.00, 2.50, and 5.00% (v/v), corresponding to final concentrations of 1, 2.5, 5, 10, 25, and 50 mg/mL (based on plant material) in the culture medium. This mixture was added to the eggs. A control group received an equal volume of ultra-pure water instead of the extract. The eggs were co-incubated with the extract for the entire 20-day culture period. Each treatment had three replicates. The dishes were incubated at 28 °C for 20 days, with brief aeration three times weekly. After incubation, egg development was assessed microscopically. The embryonation rate was calculated as: (Number of larvae-containing eggs/Total eggs examined) ×100%.

### 2.4 Animal studies

Studies on mice were reviewed and approved by the Research Ethics Committee of Yunnan Agricultural University. According to the Animal Ethics Procedures and Guidelines of the People’s Republic of China, all animals and experiments were handled strictly in accordance with good laboratory animal practice. We have complied with all relevant ethical regulations for animal use. All efforts were made to minimize animal suffering. Seventy, 8-week-old male ICR mice were randomly divided into 7 groups (*n* = 10): a blank control (no infection), a model group (infected with untreated eggs), and five experimental groups infected with eggs treated with 0.10%, 0.50%, 1.00%, 2.50%, or 5.00% *A. adenophora* extract. Treated eggs were washed and resuspended in PBS. Each mouse was orally inoculated with 0.5 mL containing 3 × 10^3^ *A. suum* eggs. Eight, days post-infection, the mice were humanely euthanised with an overdose of intraperitoneally administered sodium pentobarbital (150 mg/kg body weight) while under inhaled halothane terminal anaesthesia. Lungs were examined for gross lesions and processed using the Baermann funnel technique to recover and count migrating larvae, as the lung is the primary site of larval migration and pathology at day 8 post-infection (22, 23).

### 2.5 Transcriptome sequencing and analysis

*A. suum* eggs from the control (WH), 0.10% (EH), and 0.50% (MH) treatment groups were collected after 20 days of incubation, with three biological replicates per group. Total RNA was extracted, and cDNA libraries were constructed and sequenced on an Illumina platform by Novogene Co., Ltd. (Beijing, China). After quality control of raw reads, clean reads were mapped to the *A. suum* reference genome. RNA from eggs treated with higher concentrations (5.00%) was of insufficient quality for reliable sequencing and was therefore excluded from transcriptome analysis. Differential expression analysis was performed using DESeq2 with criteria of log_2_(FoldChange) ≥ 1 and adjusted *p*-adjust ≤0.05. Gene Ontology (GO) and Kyoto Encyclopedia of Genes and Genomes (KEGG) pathway enrichment analyses were conducted on the differentially expressed genes (DEGs).

### 2.6 Quantitative real-time PCR validation

To verify the genes related to the treatment with *A. adenophora* extract in the transcriptome results, three differentially expressed *A. suum* genes were selected for quantitative real-time PCR (qRT-PCR) validation after treatment with 0.10 and 0.50% *A. adenophora* extract, which showed significant differences in two comparison combinations (EH vs. WH, MH vs. WH). qRT-PCR analyses were performed using the SYBR ®Green Realtime PCR Master Mix (TOYOBO Co., Ltd., Osaka, Japan) according to the manufacturer’s instructions. The internal references were the actin. The reaction conditions were: an initial incubation at 95 °C for 30 s; followed by 40 cycles of 5 s (denaturation) at 95 °C, 34 s (annealing) at 55 °C and 15 s (extension) at 72 °C. The average cycle threshold value (Ct value) was used to calculate the relative expression of three expressed *A. suum* genes using the comparative 2^−△△Ct^ method. All experiments were carried out in triplicate. The primer sequences are presented in Supplementary Table S1.

### 2.7 Statistical analysis

Data on embryonation rates and larval counts are presented as mean ± standard deviation. Differences among groups were analyzed by one-way ANOVA followed by Duncan’s multiple range test using SPSS software (version 25.0). A *p*-value <0.05 was considered statistically significant.

## 3 Results

### 3.1 Effect of *A. adenophora* extract on *A. suum* egg development

**Figure 1:**
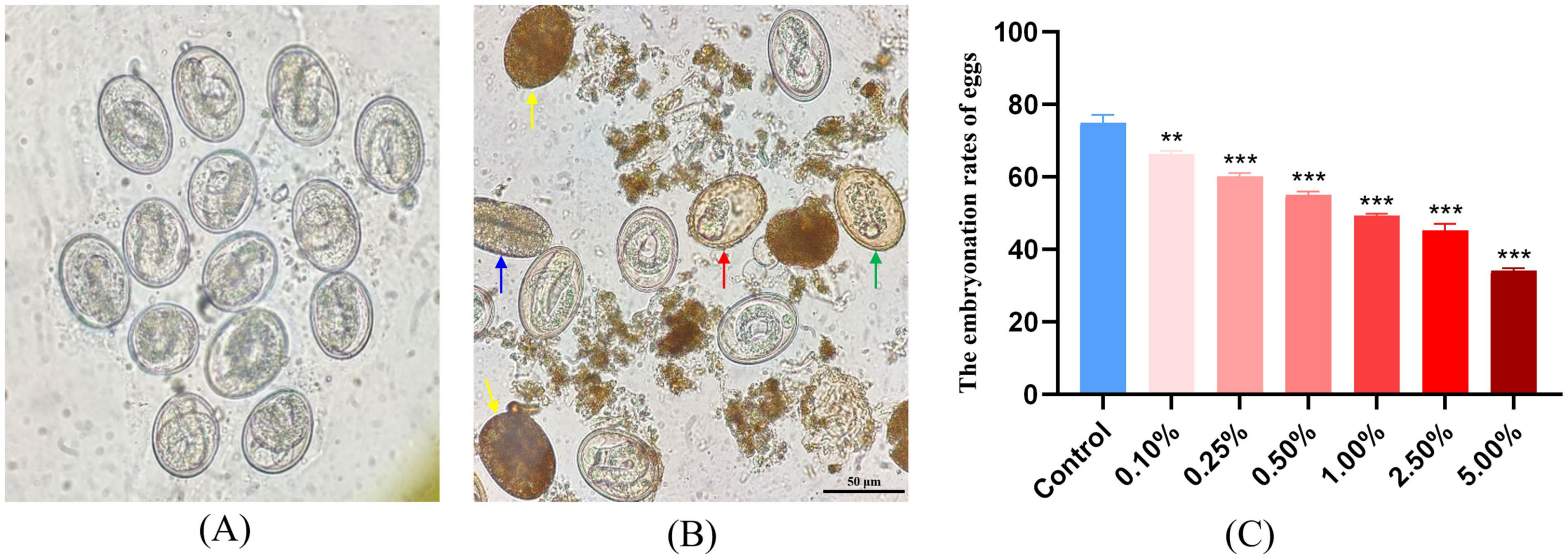
The effect of different concentrations of extracts from *A. adenophora* on the morphology and embryonation rate of *A. suum* eggs. **(A,B)** Morphology of *Ascaris suum* eggs after 20-day incubation (400×). **(A)** Control group (untreated). **(B)** 5.00% *A. adenophora* extract treatment group. Arrows indicate abnormal features: granular cytoplasm (green), vacuolization (red), darkened interior (yellow), shrinkage (blue), black scale bar: 50 μm. **(C)** The embryonation rates of *Ascaris suum* eggs after treatment with different concentrations of *A. adenophora* extract.

After exposing *A. suum* eggs to different concentrations of *A. adenophora* for 20 days, the developmental morphology of eggs from the blank control group and the 5.00% *A. adenophora* treatment group was observed under microscope. Microscopic observation revealed normal development in control eggs, with clear eggshells and larval structures (Figure 1A). In contrast, eggs treated with 5.00% extract exhibited abnormal morphology, including granular cytoplasm, vacuolization, darkened and blurred interior, and egg shrinkage (Figure 1B). In this experiment, there were differences in toxic effects between the different concentrations of *A. adenophora* extracts. After treatment with *A. adenophora*, the embryonation rate decreased significantly (*p* < 0.001) in a concentration-dependent manner, from 74.91% ± 2.18% in the control to 34.24% ± 0.56% in the 5.00% (50 mg/mL) treatment group (Figure 1C), The half-maximal inhibitory concentration (IC50) was calculated to be 4.27% (v/v), corresponding to 42.7 mg/mL.

### 3.2 Infectivity of treated *A. suum* eggs in mice

**Figure 2:**
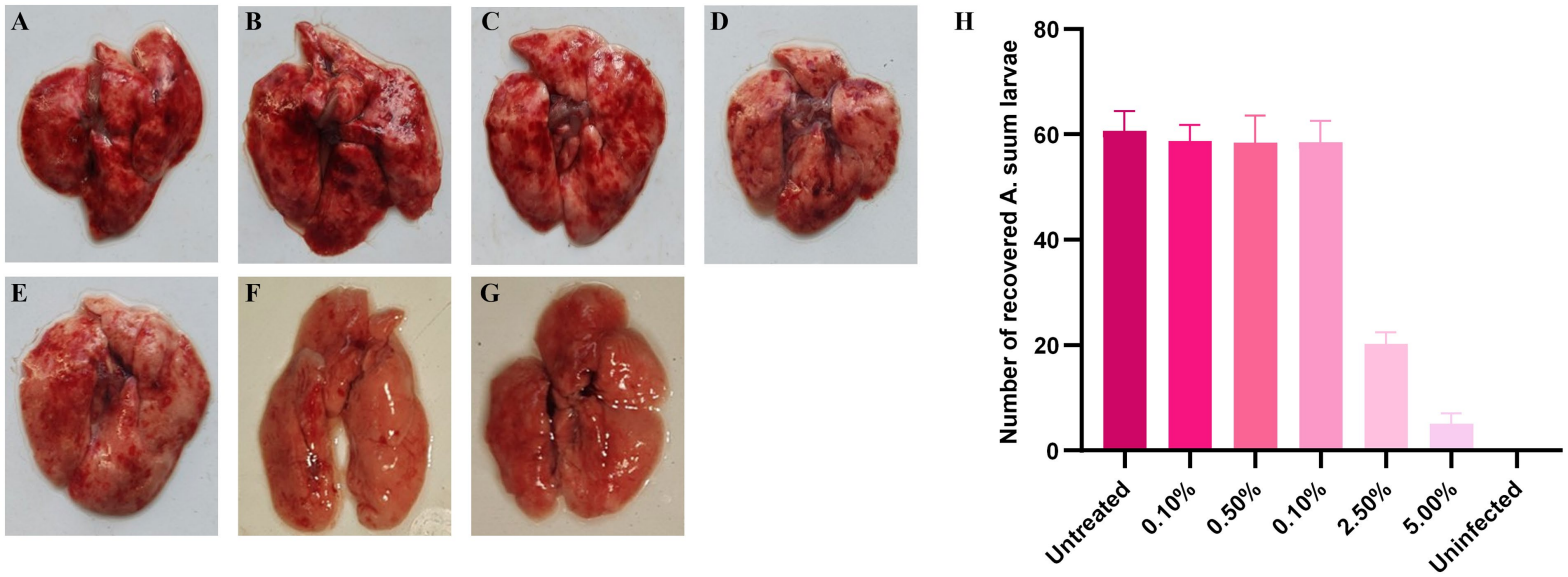
Gross pulmonary lesions in mice 8 days post-infection with *A. suum* eggs. **(A)** Model group (untreated eggs). **(B–F)** Experimental groups I–V infected with eggs treated with 0.10%, 0.50%, 1.00%, 2.50%, 5.00% *A. adenophora* extract, respectively. **(G)** Blank control group. H: Results of larval counts in organs collected by the Baermann funnel method.

Upon gross examination of the mice lung tissues, no significant lesions were observed in the blank control group. In contrast, mice inoculated with *A. suum* exhibited varying degrees of hemorrhagic lesions in the lungs. Mice infected with eggs treated with lower concentrations (0.10%–1.00%) *A. adenophora* showed severe pulmonary hemorrhage, similar to the model group (untreated eggs). Mice infected with eggs treated with 2.50% or 5.00% extract exhibited markedly reduced pulmonary lesions, with only minor petechial hemorrhages in the 5.00% group (Figure 2). Larvae were subsequently recovered from the lung tissues of mice in each group using the Baermann technique. Subsequent enumeration demonstrated that no larvae were found in the blank control group during the experiment. Larval recovery from lungs reflected this trend: high counts were obtained from the model and low-concentration treatment groups, while significantly fewer larvae (*p* < 0.05) were recovered from the 2.50% and 5.00% *A. adenophora* treated groups (Supplementary Table S2).

### 3.3 Transcriptome sequencing analysis

A total of 64.32Gb clean bases were obtained from 9 samples of RNA from untreated group (WH), 0.10% *A. adenophora* treatment group (EH), and 0.50% *A. adenophora* treatment group (MH) in the construction of the lncRNA library (Supplementary Table S2). The average GC content was 45.17%, with the proportion of filtered readings in the untreated groups (WH1, WH2, WH3) all exceeding 97.15%; The proportion of filtered readings in the 0.10% *A. adenophora* treatment group (EH1, EH2, EH3) was >97.28%, and the proportion of filtered readings in the 0.50% *A. adenophora* treatment group (MH1, MH2, MH3) was >96.39%. The average values of Q20 and Q30 were 98.71% and 96.45%, respectively. Indicating that the raw data of 9 samples have good quality and high accuracy.

**Figure 3:**
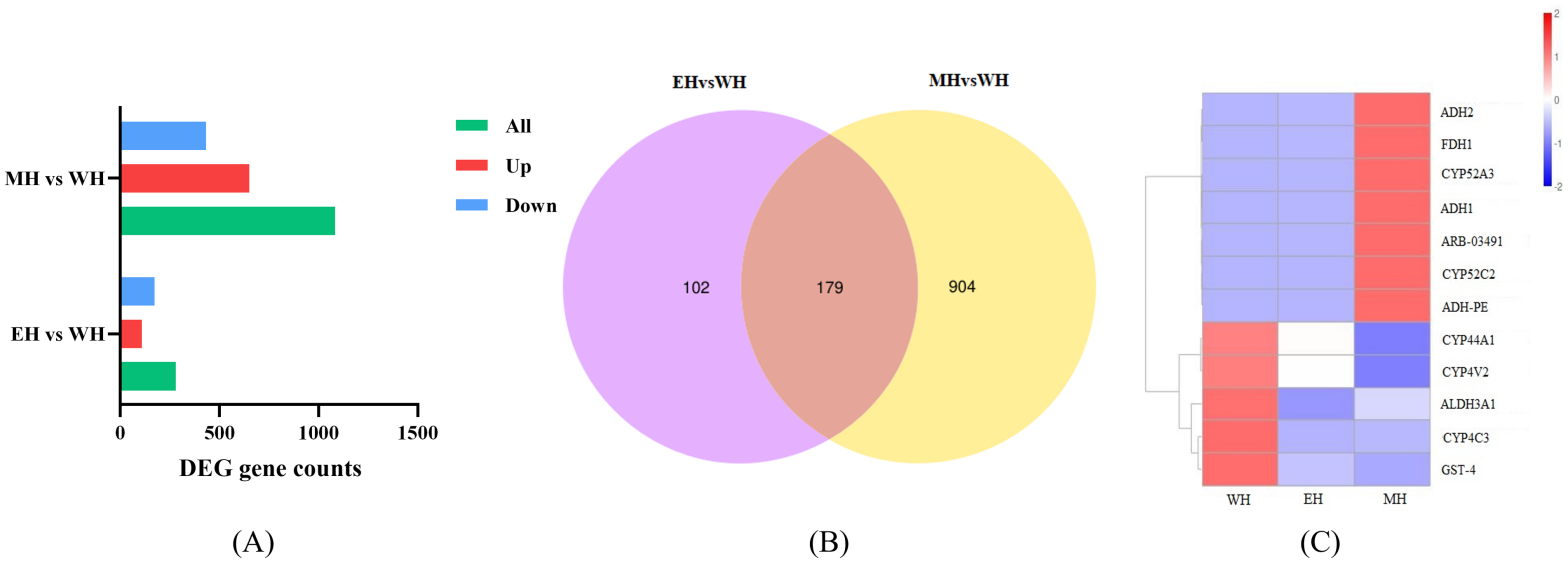
Transcriptome analysis of *A. suum* eggs. **(A)** Number of up- and down-regulated DEGs. **(B)** Venn diagram of DEGs between comparisons. **(C)** Heatmap of hierarchical clustering for selected DEGs.

According to the screening criteria, the number of differentially expressed genes (DEGs) between *A. suum* eggs treated with 0.10% *A. adenophora* extract (EH) or 0.50% extract (MH) and the untreated control group (WH) was statistically analyzed using DESeq2 with thresholds of padj ≤0.05 and |log2FoldChange| ≥ 1.0. The comparison between the EH and WH groups identified 281 DEGs, of which 109 were up-regulated and 172 were down-regulated. In contrast, the comparison between the MH and WH groups revealed a substantially higher number of DEGs, totaling 1,083 (651 up-regulated and 432 down-regulated) (Figure 3A). This result indicates that the number of DEGs increased with the rising concentration of *A. adenophora* extract.

A Venn diagram analysis of the DEGs from the two comparisons was performed (Figure 3B). The analysis showed that 179 DEGs overlapped between the EH vs. WH and MH vs. WH comparisons. Furthermore, 102 DEGs were specifically expressed in the EH vs. WH comparison, while 904 DEGs were specific to the MH vs. WH comparison.

A heatmap visualizing the expression levels of these DEGs is presented in Figure 3C. Hierarchical clustering of the DEGs revealed prominent clusters associated with genes including ADH2, FDH1, CYP52A3, ADH1, ARB-03491, CYP52C2, ADH-PE, CYP44A1, CYP4V2, ALDH3A1, CYP4C3, and GST-4. Compared with the untreated control group (WH), the expression of CYP44A1, CYP4V2, CYP4C3, and GST-4 was significantly down-regulated.

### 3.4 Biological function analysis

To further elucidate the functions of the identified DEGs, GO enrichment analysis was performed on the DEGs from the two comparison groups (EH vs. WH and MH vs. WH). A total of 84 functional GO terms were significantly enriched for the DEGs in the EH vs. WH comparison. These terms were distributed across the three main GO categories as follows: Biological Process (BP) accounted for 47.76%, Molecular Function (MF) for 27.38%, and Cellular Component (CC) for 25.00%. For the MH vs. WH comparison, 106 GO terms were significantly enriched, with a distribution of 49.06% for BP, 25.47% for MF, and 25.47% for CC.

**Figure 4:**
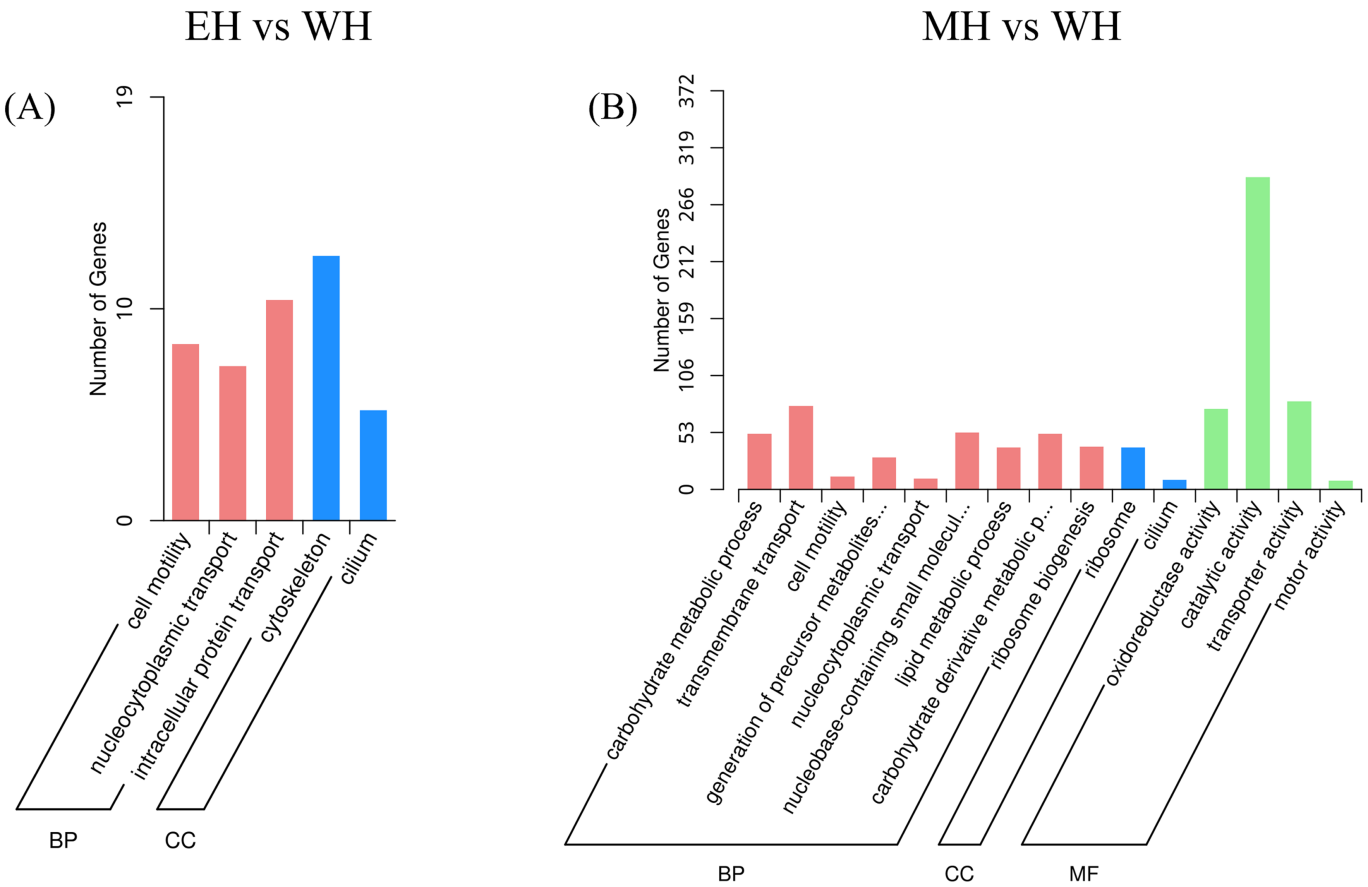
Gene ontology (GO) of DEPs between **(A)** EH and WH, **(B)** MH and WH. BP, biological process; MF, molecular function; CC, cellular component.

The top 30 significantly enriched GO terms (*p* < 0.05) for each comparison are listed in Figure 4. The DEGs from the EH vs. WH group were primarily enriched in specific BP terms such as cell motility (8 DEGs) and nucleocytoplasmic transport (7 DEGs). Within the MF category, significant enrichment was observed for motor activity (3 DEGs) and cytoskeletal protein binding (5 DEGs). For the CC category, the main enriched terms were cytoskeleton (12 DEGs) and cilium (5 DEGs) (Figure 4A).

In contrast, the GO analysis for the MH vs. WH group revealed that DEGs were predominantly enriched in BP terms related to carbohydrate metabolic process (52 DEGs), transmembrane transport (78 DEGs), and cell motility (12 DEGs). Within the MF category, the most significant enrichments were found for oxidoreductase activity (75 DEGs), catalytic activity (291 DEGs), and hydrolase activity (122 DEGs). For the CC category, the primary enriched terms were ribosome (39 DEGs) and cilium (9 DEGs) (Figure 4B).

**Figure 5:**
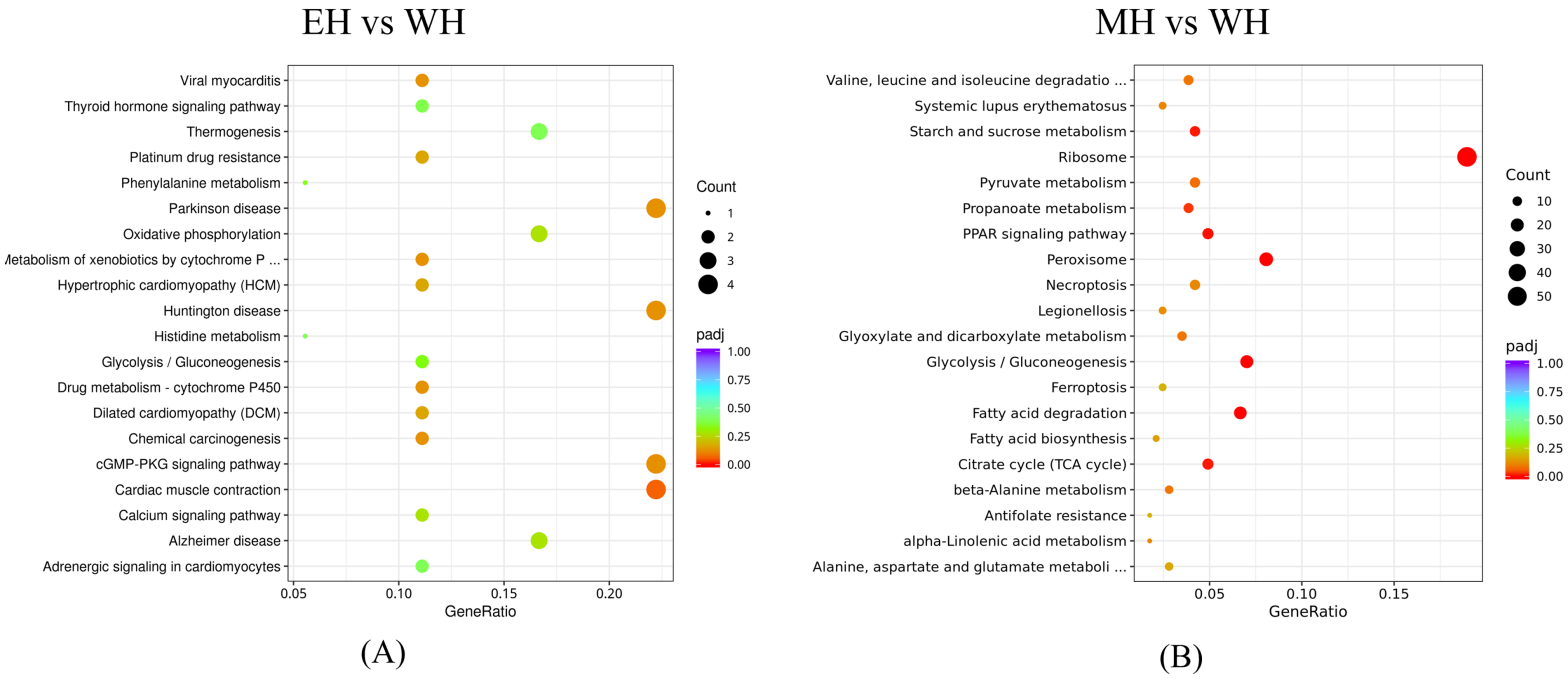
The top 20 KEGG analyses of differentially expressed genes between **(A)** EH and WH, **(B)** MH and WH. Dot size represents the number of differential genes annotated to that pathway.

Through KEGG pathway enrichment analysis, the DEGs from the EH vs. WH comparison were significantly enriched in 60 metabolic pathways, while those from the MH vs. WH comparison were enriched in a substantially greater number, totaling 249 pathways. A scatter plot of the top 20 most significantly enriched pathways for each group was generated. For the EH vs. WH comparison group, pathways such as Drug metabolism - cytochrome P450 (2 DEGs) and Glycolysis /Gluconeogenesis (2 DEGs) were among those most enriched with genes (Figure 5A). In the MH vs. WH comparison group, pathways exhibiting the highest degree of enrichment included Ribosome (54 DEGs), Glycolysis/Gluconeogenesis (20 DEGs), Valine, leucine and isoleucine degradation (11 DEGs), and Drug metabolism-cytochrome P450 (6 DEGs). Notably, both comparison groups showed significant enrichment of genes in the Drug metabolism-cytochrome P450 and Glycolysis/Gluconeogenesis pathways (Figure 5B). This result indicates that these specific metabolic pathways are substantially affected in *A. suum* eggs following exposure to *A. adenophora* extract.

### 3.5 qRT-PCR validation analysis

**Figure 6:**
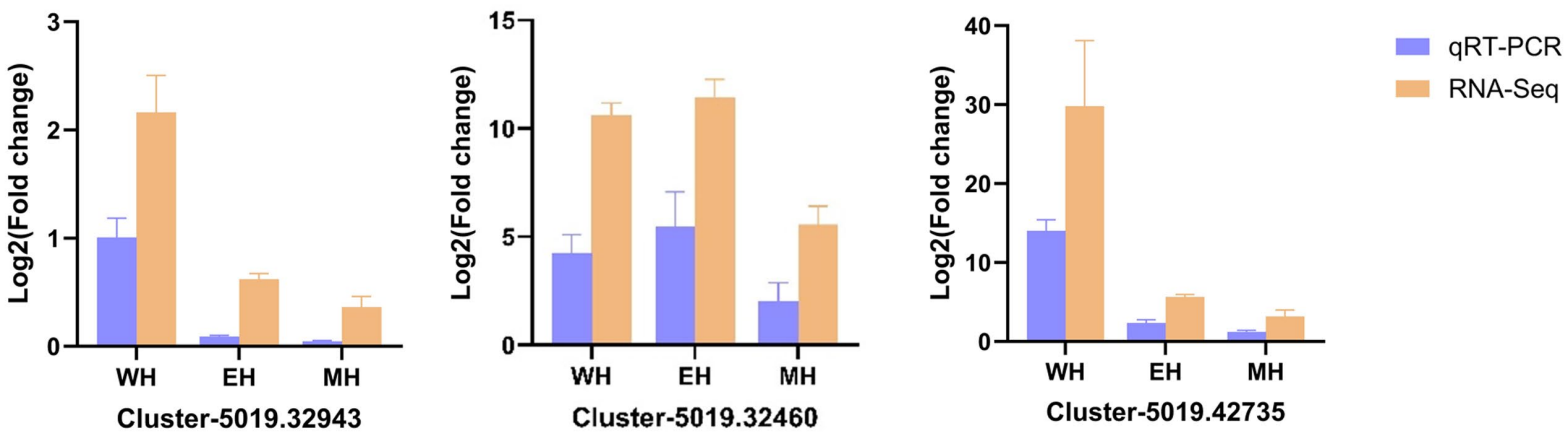
qRT-PCR validation of three differentially expressed genes from RNA-seq. Expression trends match RNA-seq results. Error bars represent SD.

To validate the accuracy and reliability of the RNA-seq data, qRT-PCRs were performed to determine the levels of expression of three significantly different genes randomly selected from two groups of RNA seq data. The results from sequencing data were in agreement with those from qRT-PCR in terms of the levels of expression of the validated differentially expressed genes (Figure 6).

## 4 Discussion

As an invasive species, *A. adenophora* is widely distributed across southwestern China, particularly in Yunnan Province, where it poses substantial ecological and economic threats to local ecosystems and livestock production (1, 2). Interestingly, this plant has also demonstrated considerable potential for insecticidal and antiparasitic applications. In this study, we investigated the effects of *A. adenophora* extracts against *A. suum* - a pathogen of major concern to the swine industry in China. Using transcriptome sequencing, we characterized the molecular responses induced by the treatment, aiming to provide a theoretical foundation for the development of novel botanical insecticides. Our study demonstrates that *A. adenophora* aqueous extract significantly inhibits the *in vitro* development and reduces the *in vivo* infectivity of *A. suum* eggs in a concentration-dependent manner. The morphological abnormalities observed in treated eggs (Figure 1) are consistent with damage caused by other toxic agents, indicating a direct ovicidal effect. The reduced larval burden and milder pathology in mice infected with eggs treated with higher concentrations (≥2.50%) further confirm that the extract compromises egg viability and subsequent larval development.

In this study, *Ascaris suum* eggs were exposed to varying concentrations of *A. adenophora* extract in culture. After 20 days of incubation, compared to the control group, most eggs treated with 5.00% glandular ginseng extract showed abnormal features, including cytoplasmic granules and vacuoles, with fuzzy internal structures. These morphological alterations are consistent with abnormalities reported by Ruisi Zhang (24) following disinfectant treatment of *A. suum* eggs, suggesting that toxic stimuli can induce comparable structural changes. Previous studies have found that the the *A. adenophora* extracts with 1.5 and 1.0 g/mL (w/v) concentrations were also toxic for *Haemaphysalis longicornis*, the median lethal time (LT50) for larval and nymphal ticks with 1.5 g/mL (w/v) concentration of extract were 0.790 (LT99 = 1.065) and 1.018 (LT99 = 10.608) hours, respectively (12). In other plants, a previous study by Challam et al. (25) showed that the alcoholic extract of *Lysimachia ramosa* Wall (*Primulaceae*) treated with *A. suum* eggs, *helminth parasites* and *Fasciolopsis buski*, respectively, resulted in varying degrees of inactivation and flaccid paralysis of the parasites, followed by death at different stages. A dose dependent loss of motility and mortality were observed in all treated parasites. Compared with other disinfectants used to treat *A. suum*, for instance, prolonged exposure to 4% ammonia solution resulted in an 81.17% egg kill rate (18.83% embryonation rate), while exposure to 1%, 5%, and 10% povidone-iodine solutions achieved 100% egg mortality (0% embryonation rate). The relatively lower anti-embryonation efficacy observed in our study may be attributed to the use of a crude plant extract, which likely contains a lower concentration of active compounds compared to refined chemical disinfectants. This difference in purity and potency could account for the diminished inhibitory effect on egg embryonation observed here. Notably, the extract significantly reduced egg infectivity in mice, as evidenced by lower larval recovery and reduced pulmonary pathology. These findings suggest that even a crude preparation has meaningful biological activity, and further purification may enhance efficacy.

The transcriptomic analysis provides molecular insights into this inhibitory effect. The dose-dependent increase in the number of DEGs suggests a profound impact of *A. adenophora* constituents on gene expression in *A. suum* eggs. Transcriptome analysis was performed only on eggs treated with 0.10% and 0.50% concentrations, as RNA from higher concentration groups (2.50% or 5.00%) was of insufficient quality due to extensive cellular damage. Consequently, the molecular analysis reflects early-stage responses rather than the full spectrum of effects at higher concentrations. Future studies using targeted approaches or single-cell transcriptomics are needed to elucidate molecular events at higher doses. GO enrichment analysis of the DEGs in *A. suum* eggs treated with *A. adenophora* extract revealed that both comparison groups shared enrichment in three major functional categories: material transport and metabolism, catalytic activity, and cytoskeleton-related functions. Notably, the number of DEGs associated with transmembrane transport within the Biological Process category increased markedly. GO enrichment points to disruptions in fundamental processes like transmembrane transport and carbohydrate metabolism. The significant enrichment of the glycolysis/gluconeogenesis pathway is particularly noteworthy, as glycolysis is a crucial energy-yielding pathway for developing embryos (26, 27). Similar to previous studies, the glycolytic metabolic activity of *A. suum* larvae exposed to Artemisinin derivatives was significantly reduced. Its disruption likely leads to an energy deficit, impairing development and embryonation.

Furthermore, the enrichment of the “Drug metabolism-cytochrome P450” pathway and the marked downregulation of cytochrome P450 genes (CYP44A1, CYP4V2, CYP4C3) and a glutathione S-transferase gene (GST-4) are key findings. Cytochrome P450 enzymes are involved in detoxifying xenobiotics and in mitochondrial electron transport (28, 29). CYP44A1, CYP4V2, and CYP4C3 are all involved in encoding members of the cytochrome P450 superfamily, their downregulation may impair detoxification of *A. adenophora* compounds and disrupt oxidative phosphorylation, reducing ATP production (30, 31). GST-4 is involved in cellular defense against oxidative stress and toxins (32). Its downregulation, similar to observations in *Caenorhabditis elegans* under toxin exposure (33, 34), may increase susceptibility to oxidative damage, leading to embryonic defects and developmental delay. The concurrent downregulation of these detoxification and metabolic genes suggests a synergistic mechanism where *A. adenophora* extract overwhelms the egg’s defense systems while crippling its energy metabolism.

Several limitations should be acknowledged in this study. First, the chemical composition of the *A. adenophora* aqueous extract was not characterized, which limits the reproducibility and identification of active compounds. Second, the mouse model, while well-established for assessing larval migration, does not fully replicate infection in the natural porcine host, and effects on adult worms were not evaluated. Third, transcriptome analysis was limited to the 0.10 and 0.50% treatment groups because RNA from higher concentrations was of insufficient quality due to extensive cellular damage, precluding reliable sequencing; thus, molecular responses at the most effective concentrations remain to be explored. Finally, the crude extract only exhibited an inhibition rate of approximately 34% on the embryonic development of *A. suum* eggs, and further purification of active constituents may enhance efficacy. Addressing these limitations in future investigations will provide a more comprehensive understanding of the anthelmintic potential of *A. adenophora*.

## 5 Conclusion

In conclusion, *A. adenophora* aqueous extract effectively inhibits the development and reduces the infectivity of *A. suum* eggs. Transcriptomic analysis indicates that this effect is mediated through the disruption of critical pathways, including energy metabolism (glycolysis) and xenobiotic detoxification (cytochrome P450 system). Key genes such as CYP44A1, CYP4V2, CYP4C3, and GST-4 are implicated in this process. These findings not only elucidate potential mechanisms of action but also provide a theoretical basis for developing *A. adenophora*-based agents targeting the egg stage of *A. suum*. Further research should focus on purifying the active constituents and evaluating their efficacy and safety in target animals.

## Data Availability

All relevant data are available in the manuscript and the Supporting Information files. The raw sequencing data have been deposited in the Genome Sequence Archive (GSA) at the National Genomics Data Center/China National Center for Bioinformation, accession number: CRA041146, https://ngdc.cncb.ac.cn/gsa/browse/CRA041146.
