# Supplementary material for: Ageratina adenophora aqueous extract impairs Ascaris suum egg embryonation and infectivity via disruption of metabolic and detoxification pathways
Source: Front Vet Sci. 2026 Apr 22;13:1789026. doi: 10.3389/fvets.2026.1789026 (PMC13147474; doi:10.3389/fvets.2026.1789026)
Supplement: Supplementary file 1 [file Table_1.docx]

Table S1 The primers of differential expression gene for RT-qPCR

| **Primers** | **Primer sequence (5'→3')** |
| --- | --- |
| Actin F | GGGTCGTGACCTCACTGATT |
| Actin R | TTGATCTTCATCGTGCTTGG |
| Cluster-5019.32943F | GTTGTATTGTTCGGCTGGTT |
| Cluster-5019.32943R | GACGGTTTGTCTTTGCTTTT |
| Cluster-5019.42735F | CTTAGCCCTGTTCGTCAA |
| Cluster-5019.42735R | GCTTTCCATCCGAGTAGTTAT |
| Cluster-5019.9739F | TGTGCCAGTCGGTAGAGT |
| Cluster-5019.9739R | TGTCACCTGGAACACCTT |

| **sample** | **library** | **raw_reads** | **raw_bases** | **clean_reads** | **clean_bases** | **error_rate** | **Q20** | **Q30** | **GC_pct** |
| --- | --- | --- | --- | --- | --- | --- | --- | --- | --- |
| WH1 | FRAS240313664-1r | 23080363 | 6.92 | 22463309 | 6.74 | 0.01 | 98.66 | 96.37 | 45.44 |
| WH2 | FRAS240313665-1r | 22656867 | 6.8 | 22010065 | 6.6 | 0.01 | 98.84 | 96.73 | 45.24 |
| WH3 | FRAS240313666-1r | 27278507 | 8.18 | 26593667 | 7.98 | 0.01 | 98.67 | 96.34 | 45.4 |
| EH1 | FRAS240313667-1r | 23878389 | 7.16 | 23228792 | 6.97 | 0.01 | 98.64 | 96.3 | 45.28 |
| EH2 | FRAS240313668-1r | 22932063 | 6.88 | 22440685 | 6.73 | 0.01 | 98.64 | 96.33 | 45.28 |
| EH3 | FRAS240313669-1r | 24655718 | 7.4 | 24078308 | 7.22 | 0.01 | 98.66 | 96.34 | 45.24 |
| MH1 | FRAS240313670-1r | 23931785 | 7.18 | 23069016 | 6.92 | 0.01 | 98.71 | 96.43 | 45.6 |
| MH2 | FRAS240313671-1r | 23959579 | 7.19 | 23675895 | 7.1 | 0.01 | 98.86 | 96.81 | 44 |
| MH3 | FRAS240313672-1r | 27214479 | 8.16 | 26867808 | 8.06 | 0.01 | 98.68 | 96.38 | 45.05 |

Table S2 Summary of sample sequencing date quality
